# Chemometric Evaluation of Urinary Steroid Hormone Levels as Potential Biomarkers of Neuroendocrine Tumors

**DOI:** 10.3390/molecules181012857

**Published:** 2013-10-16

**Authors:** Alina Plenis, Natalia Miękus, Ilona Olędzka, Tomasz Bączek, Anna Lewczuk, Zofia Woźniak, Patrycja Koszałka, Barbara Seroczyńska, Jarosław Skokowski

**Affiliations:** 1 Department of Pharmaceutical Chemistry, Medical University of Gdańsk, Hallera 107, Gdańsk 80-416, Poland; E-Mails: miekusn@gumed.edu.pl (N.M.); ilona@gumed.edu.pl (I.O.); tbaczek@gumed.edu.pl (T.B.); 2 Department of Endocrinology and Internal Medicine, Medical University of Gdańsk, Dębinki 7, Gdańsk 80-210, Poland; E-Mails: anna.lewczuk@gumed.edu.pl (A.L.); zofia.wozniak@yahoo.pl (Z.W.); 3 Department of Cell Biology, Medical University of Gdańsk, Dębinki 1, Gdańsk 80-210, Poland; E-Mail: pkosz@gumed.edu.pl; 4 Bank of Frozen Tissues and Genetic Specimen, Medical University of Gdańsk, Dębinki 1, Gdańsk 80-210, Poland; E-Mails: bastrzel@gumed.edu.pl (B.S.); chironkol@gumed.edu.pl (J.S.)

**Keywords:** neuroendocrine tumors (NETs), biomarkers, steroid hormones, HPLC determination, chemometric analysis

## Abstract

Neuroendocrine tumors (NETs) are uncommon tumors which can secrete specific hormone products such as peptides, biogenic amines and hormones. So far, the diagnosis of NETs has been difficult because most NET markers are not specific for a given tumor and none of the NET markers can be used to fulfil the criteria of high specificity and high sensitivity for the screening procedure. However, by combining the measurements of different NET markers, they become highly sensitive and specific diagnostic tests. The aim of the work was to identify whether urinary steroid hormones can be identified as potential new biomarkers of NETs, which could be used as prognostic and clinical course monitoring factors. Thus, a rapid and sensitive reversed-phase high-performance liquid chromatographic method (RP-HPLC) with UV detection has been developed for the determination of cortisol, cortisone, corticosterone, testosterone, epitestosterone and progesterone in human urine. The method has been validated for accuracy, precision, selectivity, linearity, recovery and stability. The limits of detection and quantification were 0.5 and 1 ng mL^−1^ for each steroid hormone, respectively. Linearity was confirmed within a range of 1–300 ng mL^−1^ with a correlation coefficient greater than 0.9995 for all analytes. The described method was successfully applied for the quantification of six endogenous steroid levels in human urine. Studies were performed on 20 healthy volunteers and 19 patients with NETs. Next, for better understanding of tumor biology in NETs and for checking whether steroid hormones can be used as potential biomarkers of NETs, a chemometric analysis of urinary steroid hormone levels in both data sets was performed.

## 1. Introduction

Neuroendocrine tumors (NETs) are relatively rare tumors that arise from the diffuse neuroendocrine system. About 70% of NETs derive from the gastroenterohepatic (GEP) system and the other 30% from the different sites through the body [1]. Most NETs occur as sporadic tumors or may arise in association with familial syndromes such as multiple endocrine neoplasia type 1 (MEN1), multiple endocrine neoplasia type 2 (MEN2) and von Hippel-Lindau (VHL) [2,3]. Some NETs may occasionally show very aggressive behavior and become highly malignant, but the great majority tend to be relatively slow growing and retain many multipotent differentiation capacities. Such features include the ability to produce and secrete a variety of metabolically active substances (amines and peptides) and cause distinct clinical syndromes [4]. In addition, NETs possess neuroamine uptake mechanisms and/or specific receptors at the cell membrane, which can be of great value in identifying and localizing these tumors as well as being useful in their therapy [5,6]. It has meant that diagnosis of these tumors has been aided by advances in pathological diagnosis and classification and tumor imaging with endoscopic ultrasound and somatostatin receptor fusion imaging, although surgery remains the mainstay of NET treatment [7,8,9,10]. Combining hormone measurements with tissue responsiveness, demonstrations of inappropriate secretions of insulin, gastrin, neuropeptide K, substance P, synaptophysin, parathyroid hormone (PTH) or 5-hydroxyindole-3-acetic acid (5-HIAA) as well as the application of polyclonal antibodies in RIAs of hormones, such as ACTH, insulin, and gastrin provides an increase in the diagnostic level of hormone measurements in patients with NETs [11]. Other markers, such as chromogranin A (CgA) and neuron-specific enolase (NSE) as well as peptide receptor visualization, are also of increasing importance in the diagnosis and follow-up of neuroendocrine tumors [12,13]. Unfortunately, the abovementioned tumor markers used in the diagnosis and follow-up of patients with NETs are in most instances not specific for a given tumor and circulate under normal conditions in the serum, making their use as an early diagnostic tool difficult (low sensitivity) [14,15]. Thus, investigations directed at the detection of novel tumor markers for the diagnosis and follow-up of treatment are needed. To the best of our knowledge, so far no study evaluating relationships between steroid hormone secretion and the presence of NETs has been reported. In the presented work, the prognostic possibilities of steroid profiles as potential biomarkers of NETs were evaluated. This assay was supported by bioinformatic tools like statistical tests and principal component analysis (PCA).

## 2. Results and Discussion

**Figure 1:**
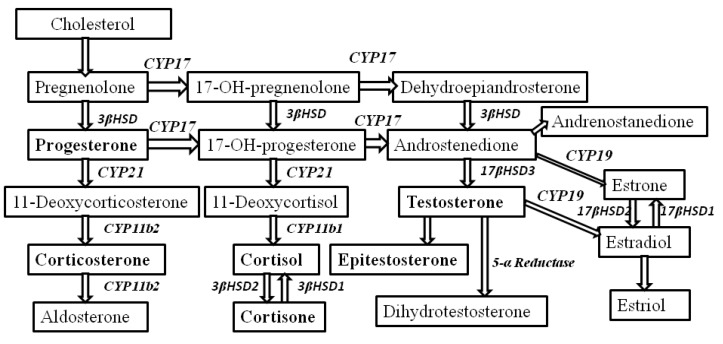
Biosynthesis of steroid hormones from the cholesterol pathway.

As mentioned above, NETs are a heterogeneous group of neoplasms that differ in their biological, chemical, and physical behaviors depending on the degree of differentiation and the location. Despite the fact that the incidence of NETs is increasing (approximately 6%/year), clinical presentation is nonspecific, resulting in delays in diagnosis (5–7 years; approximately 70% have metastases at the time of diagnosis) [16]. For instance, CgA is regarded as a major, nonspecific NET marker [17], but the results of CgA blood concentration indicate that it may actually be influenced by various factors or coexisting pathological conditions e.g., higher CgA levels in patients with diffuse disease compared with patients with local or hepatic disease were observed [18,19,20]. The biomarkers’ specificity in GEP tumors was 86% for CgA, 100% for NSE and 100% for 5-HIAA when the corresponding sensitivity was 68% for CgA, 33% for NSE and 35% for 5-HIAA, respectively [21]. It also reported that CgA and 5-HIAA together with a leading hormone in the case of functional tumors, are valuable in tumor detection, but account for 50%–90% of serological diagnosis in the case of CgA and have an even lower specificity when 5-HIAA is analyzed separately [5]. Also carcinoembryonic antigen (CEA) was found to have little diagnostic value (sensitivity of 15.4%) [22]. When the expression of neuroendocrine (NE) markers was determined by immunohistochemical staining using commercially available monoclonal antibodies it has been shown that eight percent of tumours were positive for serotonin, 18% for CD56 and 48% for NSE. Chromogranin A immunostaining was negative while only 1% of the tumours were synaptophysin immunopositive [23]. In consequence, nonfunctional NETs, lacking specific tumor markers for early detection, are often detected later in the course of the disease, and thus have a poorer prognosis. These data also indicate that our understanding of the molecular biology and mechanistic regulation of these tumors remains far from complete. For example, in the literature there are no reports describing relationships between NETs and the biosynthesis of steroid hormones from the cholesterol pathway (Figure 1) in terms of the assessment of their prognostic values for NET diagnosis.

Thus, the purpose of the study was to examine the steroid hormone profiles as potential biomarkers of NETs. To this objective, the methodology proposed in this article consisted of several stages of proceedings, including the following: (*i*) developing a rapid, simple and sensitive RP-LC method for the simultaneous determination of free cortisol, cortisone, corticosterone, testosterone, epitestosterone and progesterone in human urine; (*ii*) obtaining the profiles of six steroids for patients with NETs and healthy controls by the LC-UV method; (*iii*) the application of parametric and non-parametric tests for comparing both experimental data sets (patients with NETs *vs*. healthy controls); (*iv*) the use of principal component analysis (PCA) for presenting the structure of variables and objects in a three-dimensional space which offers the possibility of a deeper interpretation of the obtained results. A more detailed description of the following steps of the study has been presented below.

### 2.1. LC-UV Method for the Simultaneous Determination of Six Steroid Hormones in Human Urine

In the literature there are many analytical methods for the quantification of steroids in human urine [24,25,26,27,28,29,30,31,32,33,34,35,36,37,38,39,40]. Unfortunately, a lot of them possess various limitations, like cross-reactivity with other endogenous steroids [30,37,38], a complicated derivatization procedure [25,26,27], long sample preparation procedure [35,36] or high detection limits [24,28,34,39]. This means that these methodologies may be problematic for routine steroid analysis in clinical laboratories. Thus, a challenge for years to come is the development of a simple and sensitive LC method for the simultaneous determination of steroid hormones in a biological matrix in view of diagnostic as well as biomedical and pharmacokinetic studies.

#### 2.1.1. Optimization of Sample Preparation

All the details concerning the optimization of urinary sample preparation were described in the previous paper [32].

#### 2.1.2. Optimization of LC Parameters

The proposed LC method was fully optimized to provide a simple, sensitive and accurate procedure for the quantification of six steroid hormones in human urine samples. Thus, many LC experiments were carefully performed prior to the final selection of an appropriate analytical column, a mobile phase composition, a program of gradient elution and a flow-rate as well as an optimal temperature and a wavelength for UV detection. The best LC separation of six steroids in urine samples was obtained on a Discovery HS column (250 × 4 mm, 5 µm) using gradient elution of the mobile phase composed with water (solvent A) and a binary mixture of acetonitrile and water (50:50, *v/v*). The gradient program was described in detail in the part: LC conditions. An optimal flow-rate of 1 mL min^−1^ and a temperature of 40 °C for the chromatographic separation of the tested analytes were chosen taking into account the compromise between the shortest retention times and the best peak separation. Moreover, all steroid hormones were monitored at 240 nm, and a volume injection of 30 µL was selected as optimal for further analysis for the improvement of the sensitivity of the method and good resolution for the analytes.

#### 2.1.3. Validation of the Method

The method was fully validated in accordance with the FDA and ICH recommendations. Standard samples for the construction of calibration curves, quality control samples (QC), blank urine samples and further, unknown urine samples were extracted using the SPE technique, and analyzed in the LC conditions described in part 3.3.. Calibration curves ranging from 1 to 300 ng mL^−1^ for each analyte were constructed by plotting the peak area ratios of the analyte to the I.S. (internal standard) against the steroid concentration in ng mL^−1^ using a least-squares linear regression model.

Linearity was checked on the basis of calibration curves using spiked blank urine samples in order to obtain eight different concentrations (1, 5, 10, 20, 50, 100, 200 and 300 ng mL^−1^), and the I.S. at a level of 100 ng mL^−^^1^*.* These calibration samples were processed and analyzed on the same day. The correlation coefficients (*R^2^*) were found to be more than 0.9995 for all compounds of interest, which indicates the excellent linearity of the method in the considered concentration range (Table 1).

**Table 1:** Summary of validation data within a range of 1–300 ng mL^−1^ for six steroid hormones in human urine obtained with LC calibrations (n = 6).

Steroids	Equation parameter		Standard error	Correlation coefficient (*R^2^*)	LOD [ng mL^−1^]	LOQ [ng mL^−1^]
	Slope	Intercept				
Cortisone	0.0037 ± 0.00001	−0.0033 ± 0.0026	0.0056	0.9998	0.5	1
Cortisol	0.0019 ± 0.00001	0.0017 ± 0.0023	0.0050	0.9995		
Corticosterone	0.0060 ± 0.00004	0.0018 ± 0.0055	0.0121	0.9997		
Testosterone	0.0082 ± 0.00005	0.0007 ± 0.0069	0.0150	0.9998		
Epitestosterone	0.0097 ± 0.00005	0.0135 ± 0.0076	0.0166	0.9998		
Progesterone	0.0094 ± 0.00006	−0.0009 ± 0.0086	0.0187	0.9997		

**Figure 2:**
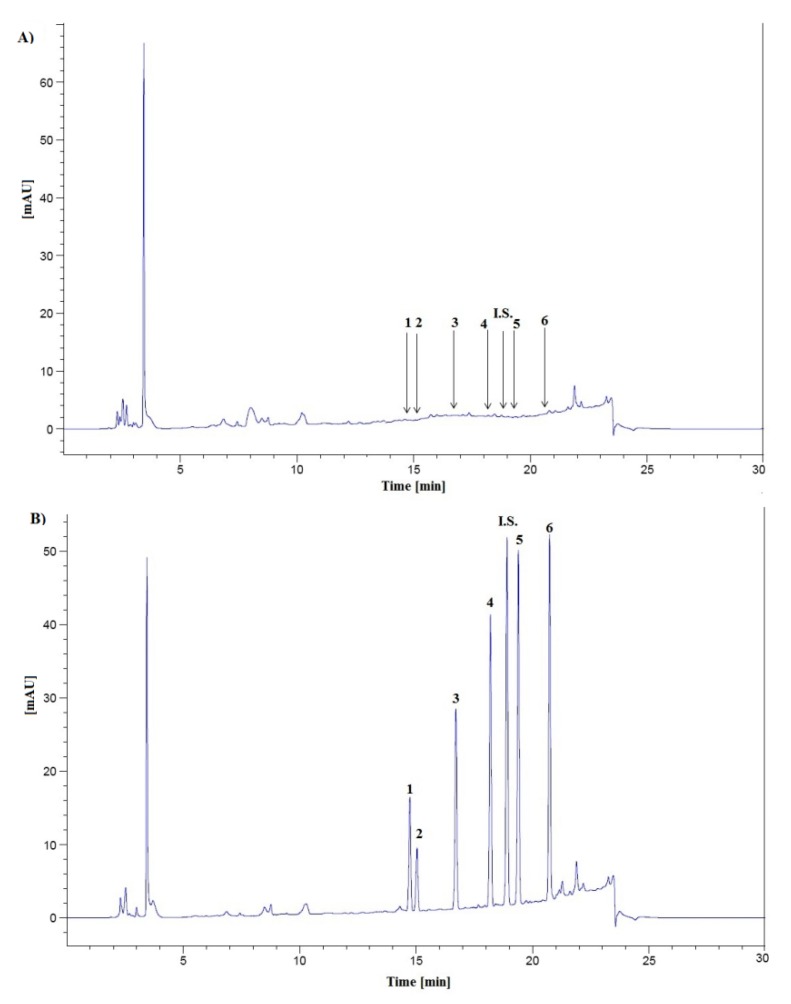
Typical chromatograms of blank human urine extract (**A**) and urine sample spiked with cortisone (**1**); cortisol (**2**); corticosterone (**3**); testosterone (**4**); epitestosterone (**5**); progesterone (**6**), each analyte at a level of 100 ng mL^−1^ and methyltestosterone (I.S.) at a concentration of 100 ng mL^−1^ (**B**).

The selectivity of the method was determined by analyzing blank and spiked urine samples from different donors to test for interference from peaks at the retention times of the compounds of interest (n = 6). Typical chromatograms of blank extract and urine extract sample spiked with six hormones, each analyte at a concentration of 100 ng mL^−1^ and I.S. at a level of 100 ng mL^−1^ are shown in Figure 2A,B respectively. No interference with constituents from urine samples was observed.

For each analyte, the limit of detection (LOD) was calculated from six independently made replications, and was determined as the lowest measurable sample concentration at which the peak, three times that of the baseline noise, was calculated. The limit of quantification (LOQ) was defined as the lowest concentration which can be detected with the precision expressed by relative standard deviations (RSD%) below 15%, accuracy expressed as a percent of the nominal concentrations within 80%–120%, and a ratio of signal- to-noise better than 10. For each hormone, the LOD was experimentally set at 0.5 ng mL^−1^ while the LOQ was also taken experimentally as the lowest concentration on the calibration curve and set at 1 ng mL^−1^, respectively. The method was sufficiently sensitive, with LOQ values comparable or lower than for the earlier published HPLC methods [24,28,31,32,33,34,35,39].

Intra- and inter-day precision and accuracy were assessed by analyzing the quality control (QC) samples (20, 50 and 100 ng mL^−1^ for cortisol and cortisone, and 5, 20 and 50 ng mL^−1^ for corticosterone, testosterone, epitestosterone and progesterone, respectively) which were determined by sixfold replicate analyses during the same day (intra-day variability) and six different days within 1 month (inter-day variability), respectively. The results are summarized in Table 2. As shown, the intra- and inter-day precisions, expressed as the RSD%, were below 8.4% and 9.8%, respectively. The intra- and inter-day accuracies calculated by assessing the agreement between the measured and known concentration of analyzed samples [(found/added amount) × 100%] were above 98.2% and 93.9%, respectively.

The absolute recovery of each steroid in urine sample was determined at two concentration levels (10 and 100 ng mL^−1^) with six replicates for each concentration. Mean absolute recoveries of the analytes were above 96.1%. The absolute recovery of the internal standard (methyltestosterone) was 98.9% ± 2.5%. The proposed SPE procedure with an HLB cartridge offers adequate sensitivity for the evaluation of free endogenous steroid levels in human urine samples.

**Table 2:** Intra-day and inter-day precision and accuracy for the LC determination of six steroid hormones in urine samples.

Concentration (ng mL^−1^)		Intra-day (n = 6)		Concentration (ng mL^−1^)	Inter-day (n = 6)		Absolute recovery
Spiked (ng mL^−1^)	Found (mean ± SD)	Precision RSD (%)	Accuracy (%)	Found (mean ± SD)	Precision RSD (%)	Accuracy( %)	(%)
Cortisone							
20	20.2 ± 1.3	6.4	101.1	19.4 ± 1.3	6.7	97.0	
50	52.2 ± 2.4	4.6	104.5	50.9 ± 2.4	4.7	101.9	99.2 ± 3.1
100	98.2 ± 3.4	3.5	98.2	99.7 ± 1.2	1.2	99.7	
Cortisol							
20	20.5 ± 1.4	6.8	102.5	19.0 ± 1.8	9.5	95.1	
50	51.8 ± 3.3	6.4	103.6	52.0 ± 3.5	6.7	104.1	96.7 ± 4.3
100	104.4 ± 4.7	4.5	104.4	99.9 ± 2.8	2.8	99.9	
Corticosterone							
5	5.2 ± 0.4	7.6	104.0	4.7 ± 0.4	8.5	93.9	
20	21.4 ± 1.5	6.9	106.8	20.5 ± 1.4	6.8	102.3	96.1 ± 4.8
50	49.7 ± 2.9	6.0	99.4	49.9 ± 1.2	2.4	99.7	
Testosterone							
5	5.2 ± 0.4	8.4	104.0	4.8 ± 0.4	8.3	95.6	
20	20.6 ± 1.5	7.5	103.4	20.3 ± 1.1	5.4	101.5	97.8 ± 3.4
50	49.9 ± 3.1	6.4	99.8	49.8 ± 0.9	1.8	99.7	
Epitestosterone							
5	5.1 ± 0.4	7.2	101.7	5.1 ± 0.5	9.8	101.5	
20	21.3 ± 0.9	4.3	106.4	19.9 ± 1.1	5.5	99.6	98.8 ± 5.2
50	51.2 ± 1.6	3.1	102.4	50.4 ± 0.9	1.7	100.2	
Progesterone							
5	5.4 ± 0.4	7.4	107.9	5.2 ± 0.5	9.6	103.2	
20	20.3 ± 0.9	4.7	101.5	19.8 ± 1.0	5.0	99.0	97.5 ± 4.7
50	50.1 ± 2.3	4.6	100.2	50.1 ± 0.8	1.6	100.3	

The obtained validation data confirmed that each analyte met the generally accepted criteria for bioanalytical method validation recommended by the FDA and ICH guidelines.

As part of the validation, the freeze-thaw stability of steroid hormones in urine samples was tested by measuring three replicates at each QC concentration level for each steroid during two months. The results confirm that the analytes are stable in investigated human urine samples for three cycles of freeze (−20 °C) and thaw to room temperature and could be handled under normal laboratory conditions without significant loss (data not shown).

### 2.2. Application of the Method in Real Human Urine Samples

The urinary steroid hormone profiles in humans reflect the diurnal variation of the plasma concentrations. On the other hand, the significant urinary steroid differences are also associated with diurnal and monthly cycles of hormone secretion [41,42]. To avoid the diurnal fluctuation of the steroids, 24-hour urine specimens from the subjects were taken into account during the study. Moreover, urinary creatinine concentrations were measured for all subjects participating in the study because the increase or decrease of the hormone levels may be associated with kidney function efficiency. Additionally, abnormal physiological creatinine levels in urine may signal renal failure and/or a reduced glomerular filtration. The mean urinary creatinine levels ranged from 0.91 to 1.03 mg dL^−1^ for healthy controls and between 0.94 and 1.24 mg dL^−1^ for patients with NETs. This confirms that kidney dysfunction for all tested participants was excluded.

**Figure 3:**
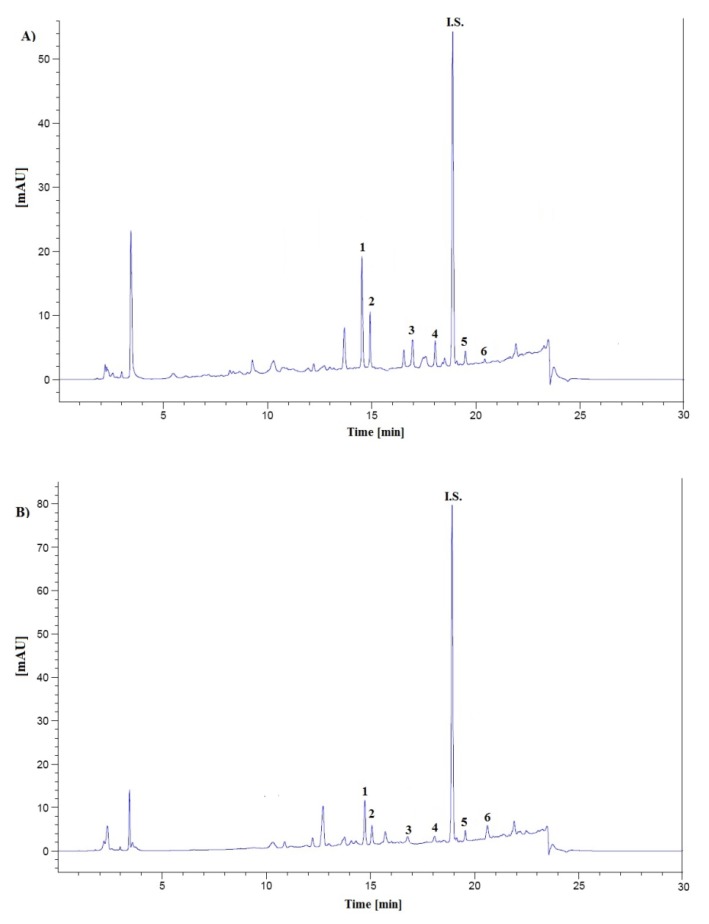
Representative LC chromatograms of human urine extract obtained from a healthy control (male) (**A**) and a patient with NETs (female) (**B**) at the level of cortisone (**1**); cortisol (**2**); corticosterone (**3**); testosterone (**4**); epitestosterone (**5**); progesterone (**6**): A—111.2; 108.1; 34.1; 11.6; 2.1; 1.4 ng mL^−1^; B—57.1; 35.9; 2.8; 3.7; 1,4; 14.4 ng mL^−1^, respectively and methyltestosterone (I.S.) at a concentration of 100 ng mL^−1^.

Next, for all subjects the profiles of six steroid hormones in urine samples were determined. Representative chromatograms of urine extract from healthy volunteers (A), and patients with NETs are shown in Figure 3A,B, respectively.

In Table 3 the results for all subjects are summarized, expressed as mean ± SD in ng mL^−1^ (the 24-h urinary sample volume was taken into account for each subject). The data indicate that the mean levels of urinary cortisone, cortisol, and corticosterone for healthy volunteers were 135.1 ± 62.8; 74.8 ± 52.6 and 10.2 ± 9.9 ng mL^−1^ whereas the mean testosterone, epitestosterone and progesterone concentrations were 9.9 ± 10.3; 2.9 ± 0.7 and 3.8 ± 2.7 ng mL^−1^, respectively. These results were comparable to the data presented in previously reported articles [28,31,39,40]. Moreover, urinary steroid levels for the men and women studied were also separately calculated. The obtained results indicated that higher mean concentrations of cortisol and progesterone were observed for healthy women than healthy men. On the other hand, lower urinary cortisone and testosterone levels were determined for women in comparison to men whereas corticosterone and epitestosterone levels for both groups were comparable. In the case of all patients with NETs, the mean cortisone, cortisol and corticosterone concentrations were clearly lower in comparison to healthy controls while the urinary profiles of other tested hormones were comparable. Moreover, higher differences of urinary cortisol levels were observed between women with NETs and healthy women (23.5 ± 17.6 *vs*. 85.0 ± 69.3 ng mL^−1^) than those calculated for men from both analyzed groups (35.5 ± 52.1 *vs*. 64.4 ± 28.4 ng mL^−1^). In the case of cortisone profiles, these differences were comparable for both men and women (55.4 ± 21.3 *vs*. 146.7 ± 62.6 ng mL^−1^, and 46.5 ± 19.3 *vs*. 123.6 ± 64.2 ng mL^−1^, respectively) whereas corticosterone concentrations were lower for women with NETs. Other urinary steroid profiles established for both analyzed groups were comparable.

**Table 3:** The results of six urinary free steroid levels measured by the LC-UV method in healthy controls against patients with NETs.

Subjects	Urinary concentration of the hormones (ng mL^−1^) (mean ± SD)					
	Cortisone	Cortisol	Corticosterone	Testosterone	Epitestosterone	Progesterone
Healthy controls (HC)						
All; n = 20	135.1 ± 62.8	74.8 ± 52.6	10.2 ± 9.9	9.9 ± 10.3	2.9 ± 0.7	3.8 ± 2.7
Females (HF); n = 10	123.6 ± 64.2	85.0 ± 69.3	10.9 ± 10.8	3.6 ± 1.0	2.8 ± 0.8	6.0 ± 2.0
Males (HM); n = 10	146.7 ± 62.6	64.7 ± 28.4	9.5 ± 9.5	16.2 ±11.5	3.0 ± 0.5	1.6 ± 1.1
Patiens with NET (P)						
All; n = 19	51.2 ± 20.3	29.8 ± 39.2	6.2 ± 4.0	9.7 ± 8.3	3.4 ± 4.7	4.8 ± 4.7
Females (PF); n = 9	46.5 ± 19.3	23.5 ± 17.6	4.3 ± 2.1	3.8 ± 2.2	2.0 ± 1.1	8.1 ± 5.0
Males (PM); n = 10	55.4 ± 21.3	35.5 ± 52.1	7.9 ± 4.5	14.9 ± 8.4	4.7 ± 6.2	1.8 ± 0.9

Initially, the data for cortisol and cortisone levels calculated for the patients with NETs and the healthy controls seem surprising. According to the data reported in many papers the secretion of cortisol in humans is increased in situations of stress [32,39,43]. Thus, higher glucocorticoid concentrations should theoretically be observed for patients with NETs. On the other hand, 11 out of 19 patients with NETs were treated with an analogue of somatostatin which may reduce of steroid secretion by suppressing ACTH. An interesting fact is also that the analogues of somatostatin are not used in the therapy of ACTH hyperactivity because the effectiveness of this therapy was poor [44,45]. Perhaps, not only somatostatin therapy is responsible for the differences observed in the profiles of cortisol and cortisone in patients with NETs.

### 2.3. Parametric and Non-Parametric Test Results

Parametric and non-parametric tests were used for checking whether the differences between urinary steroid levels obtained from the whole group of patients with NETs and healthy volunteers (both men and women), as well as the concentration variations observed between women and men determined within the analyzed groups were statistically significant. During the statistical analysis, the mean values, the ranges and the standard deviations of the corresponding experimental data sets were taken into account. Thus, the urinary profiles of six analyzed steroids for all the tested subjects were divided into six groups: healthy controls, both females and males (HC), healthy males (HM); healthy females (HF); patients with NETs, both males and females (P); patients with NETs, males (PM); patients with NETs, females (PF) and compared using the appropriate parametric or non-parametric tests.

The statistical analysis based on parametric and non-parametric tests was performed according to the rules presented in part 3.6.1. Firstly, the normal distribution using the Shapiro-Wilk test and the homogeneity of variance by the Brown-Forsythe test were calculated for six steroid profiles determined for all the analyzed groups. Next, according to the obtained results, a parametric analysis of the variables based on the Student’s *t*-test or the separate-variances *t* test was performed. Moreover, a non-parametric Mann-Whitney U test was applied when the steroid profiles had not met the criteria of normal distribution.

As it can be noticed in Table 4, statistically significant differences of cortisone level (*p* < 0.05) were confirmed between PM *vs*. HM; PF *vs*. HM; HC *vs*. PM; HC *vs*. PF; HF *vs*. PM; HF *vs*. PF as well as P *vs*. HM; P *vs*. HC; and P *vs*. HF. The urinary cortisol levels established between PM and HM, PF and HM, HC and PM, HF and PM, HF and PF as well as P and HM, P and HC, P and HF were also significantly different. This may suggest that the metabolism of cortisol can be changed by NET activity.

Of course, as mentioned above, most of the patients with NETs were treated with somatostatin analogues decreasing the secretion of the hormones. On the other hand, the differences of cortisone and cortisol concentrations observed between HC and P as well as within these groups (HF *vs*. PF and PM *vs*. PF, respectively) may suggest that other factors can also be responsible for these statistically significant differences. Thus, the possibility of the use of cortisone and cortisol profiles as potential biomarkers of NETs, in particular during follow up, should be considered. This proposition can be based on the fact that a more advanced stage of the disease, shorter progression free survival, and/or overall survival was recorded for study patients who had lower cortisol concentrations. None of the study participants had metastases to the hypophysis or to the adrenal glands. No significant differences of corticosterone concentrations in urine samples were calculated for any of the analyzed groups.

In the case of testosterone, these profiles were significantly different between PF and HM, PF *vs*. PM, HC *vs*. HM, HC *vs*. PM, HC *vs*. PF, HF *vs*. HM, HF *vs*. PM, HF *vs*. HC as well as P *vs*. PF. This can be easily explained by gender relationships. On the other hand, testosterone levels determined between HF and PF, HM and PM as well as HC and P were not statistically different. Thus, the influence of NETs on the synthesis and metabolism of this hormone was not confirmed.

**Table 4:** Results of parametric and non-parametric calculations using the Student’s *t*-test, the separate*-*variances *t* test and the Mann-Whitney U test, respectively (statistically significant differences are indicated in bold).

CORTISONE														
				HM		PM		PF		HC	HF			P	
**HM**						**0.0004 ^a^**		**0.0002 ^a^**		0.6383 ^a^	0.4257 ^a^			**0.0008 ^b^**	
**PM**				**0.0004 ^a^**				0.3531 ^a^		**0.00002 ^b^**	**0.0086 ^b^**			0.6042 ^a^	
**PF**				**0.0002 ^a^**		0.3531 ^a^				**0.000005 ^b^**	**0.0041 ^b^**			0.5662 ^a^	
**HC**				0.6383 ^a^		**0.00002 ^b^**		**0.000005 ^b^**			0.6409 ^a^			**0.000009 ^b^**	
**HF**				0.4257 ^a^		**0.0086 ^b^**		**0.0041 ^b^**		0.6409 ^a^				**0.0059 ^b^**	
**P**				**0.0008 ^b^**		0.6042 ^a^		0.5662 ^a^		**0.000009 ^b^**	**0.0059 ^b^**				
**CORTISOL**														
				**HM**		**PM**		**PF**		**HC**	**HF**			**P**	
**HM**						**0.0091 ^c^**		**0.0016 ^a^**		0.9824 ^c^	0.4082 ^b^			**0.0009** ^c^	
**PM**				**0.0091 ^c^**				0.7133 ^c^		**0.0025 ^c^**	**0.0113 ^c^**			0.8364 ^c^	
**PF**				****0.0016 ^a^****		0.7133 ^c^				0.8248 ^c^	**0.0214 ^b^**			0.8248 ^c^	
**HC**				0.9824 ^c^		**0.0025 ^c^**		0.8248 ^c^			0.9824 ^c^			**0.0001 ^c^**	
**HF**				0.4082 ^b^		**0.0113 ^c^**		**0.0214 ^b^**		0.9824 ^c^				**0.0035 ^c^**	
**P**				**0.0009 ^c^**		0.8364 ^c^		0.8248 ^c^		**0.0001 ^c^**	**0.0035 ^c^**				
**CORTICOSTERONE**														
				**HM**		**PM**		**PF**		**HC**	**HF**			**P**	
**HM**						0.9097 ^c^		0.0942 ^c^		0.9824 ^c^	0.9698 ^c^			0.3959 ^c^	
**PM**				0.9097 ^c^				0.0791 ^c^		0.8430 ^c^	0.8501 ^c^			0.3238 ^c^	
**PF**				0.0942 ^c^		0.0791 ^c^				0.2902 ^c^	0.2364 ^c^			0.2902 ^c^	
**HC**				0.9824 ^c^		0.8430 ^c^		0.2902 ^c^			0.9824 ^c^			0.3914 ^c^	
**HF**				0.9698 ^c^		0.8501 ^c^		0.2364 ^c^		0.9824 ^c^				0.5977 ^c^	
**P**				0.3959 ^c^		0.3238 ^c^		0.2902 ^c^		0.3914 ^c^	0.5977 ^c^			
**TESTOSTERONE**														
				**HM**		**PM**		**PF**		**HC**	**HF**			**P**
**HM**						0.0708 ^a^		**0.0004 ^c^**		**0.0292 ^c^**	**0.0070 ^b^**			0.3959 ^c^
**PM**				0.0708 ^a^				**0.0007 ^c^**		**0.0294 ^c^**	**0.0021 ^b^**			0.0568 ^c^
**PF**				**0.0004 ^c^**		**0.0007 ^c^**				**0.0412 ^c^**	0.5956 ^c^			**0.0412 ^c^**
**HC**				**0.0292 ^c^**		**0.0294 ^c^**		**0.0412 ^c^**			**0.0294 ^c^**			0.9887 ^c^
**HF**				**0.0070 ^b^**		**0.0021 ^b^**		0.5956 ^c^		**0.0294 ^c^**				0.0511 ^c^
**P**				0.3959 ^c^		0.0568 ^c^		**0.0412 ^c^**		0.9887 ^c^	0.0511 ^c^			
**EPITESTOSTERONE**														
				**HM**		**PM**		**PF**		**HC**	**HF**			**P**
**HM**						0.3846 ^c^		**0.0484 ^b^**		0.9824 ^c^	0.9983 ^a^			**0.0459 ^c^**
**PM**				0.3846 ^c^				0.1779 ^c^		0.4157 ^c^	0.6231 ^c^			0.4490 ^c^
**PF**				**0.0484 ^b^**		0.1779 ^c^				**0.0121 ^a^**	0.0636 ^a^			0.4169 ^c^
**HC**				0.9824 ^c^		0.4157 ^c^		**0.0121 ^a^**			0.9991 ^a^			**0.0476 ^c^**
**HF**				0.9983 ^a^		0.6231 ^c^		0.0636 ^a^		0.9991 ^a^				0.1483 ^c^
**P**				**0.0459 ^c^**		0.4490 ^c^		0.4169 ^c^		**0.0476 ^c^**	0.1483 ^c^			
**PROGESTERONE**														
				**HM**		**PM**		**PF**		**HC**	**HF**			**P**
**HM**						0.6134 ^a^		**0.0054 ^b^**		**0.0046 ^b^**	**0.00001 ^a^**			0.0773 ^c^
**PM**				0.6134 ^a^				**0.0054 ^b^**		**0.0078 ^b^**	**0.00006 ^b^**			0.0511 ^c^
**PF**				**0.0054 ^b^**		**0.0054 ^b^**				**0.0344 ^b^**	0.2218 ^a^			**0.0365 ^c^**
**HC**				**0.0046 ^b^**		**0.0078 ^b^**		**0.0344 ^b^**			**0.0344 ^a^**			0.8112 ^c^
**HF**				**0.00001 ^a^**		**0.00006 ^b^**		0.2218 ^a^		**0.0344 ^a^**				0.0511 ^c^
**P**				0.0773 ^c^		0.0511 ^c^		**0.0365 ^c^**		0.8112 ^c^	0.0511 ^c^			

^a^—Student’s *t*-test; ^b^—the separate*-*variances *t* test; ^c^—the Mann-Withney U test; HM—Healthy volunteers, males; PM—Patients with NET, males; PF—Patients with NET, females; HC—Healthy controls (females and males); HF—Healthy volunteers, females; P—Patients with NET (females and males).

Epitestosterone levels were significantly different between HC and P, which may suggest that this hormone can be considered as a potential biomarker of NETs. On the other hand, the differences between HM and PM as well as HF against PF were not calculated as significant. Thus, further study in terms of evaluating the relationship between NET disease and epitestosterone concentrations should be continued.

A similar situation was also observed for progesterone. Thus, progesterone profiles were statistically different between men and women from both analyzed groups (PF *vs*. HM, PF *vs*. PM, HC *vs*. HM, HC *vs*. PM, HC *vs*. PF, HF *vs*. HM, HF *vs*. PM, HF *vs*. HC, and P *vs*. PF), but these differences were not statistically significant between PM against HM, HF *vs*. PF, and P *vs*. HC, respectively. Thus, no influence of NET disorders was confirmed on progesterone secretion for men and women.

### 2.4. Principal Component Results

**Figure 4:**
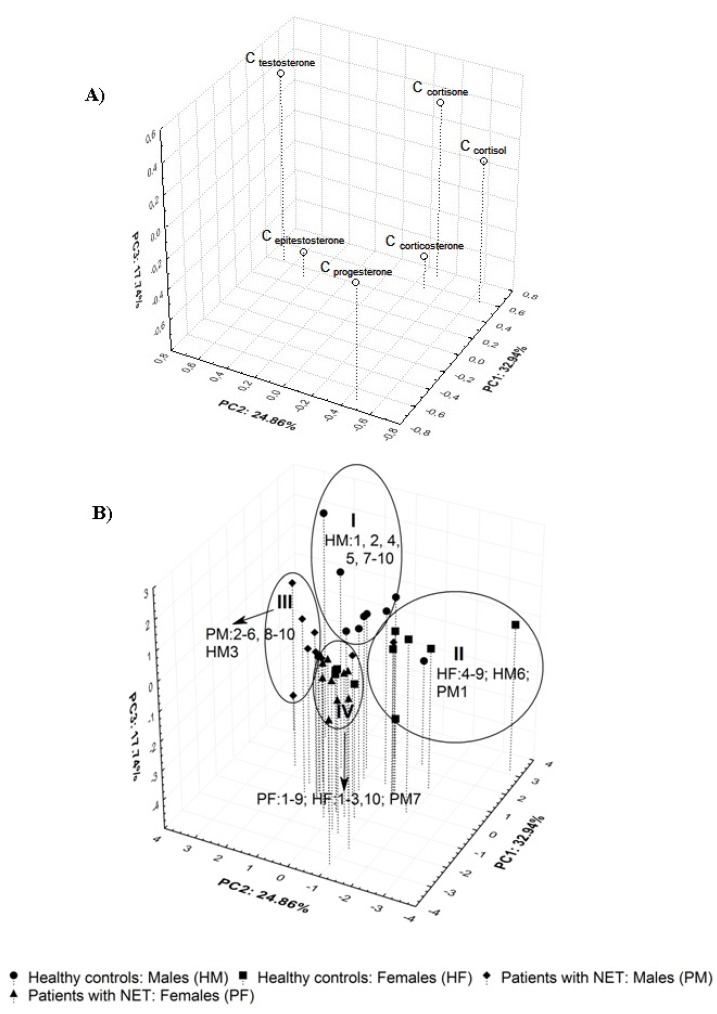
The projection of the variables (**A**) and the subjects (**B**) into a three-dimensional space created by PCA based on six urinary free steroid profiles determined for the investigated groups.

Principal component analysis (PCA) offering the possibility of a graphic data visualization of the relationships between the variables and/or the objects without losing any significant information was also conducted for checking whether steroid hormone profiles in human urine could be used to facilitate the prognosis in NET patients. The three-dimensional loadings and score plots for the variables and objects derived from the urinary concentrations of six steroid hormones in the 39 participants studied are illustrated in Figure 4A,B respectively. Notably, the first principal components (PC1) explaining 32.94% of the variance of the analyzed data were related mainly with the variability of cortisone and progesterone levels. These variables were located on opposite sides of the PC1 axes (Figure 4A). The variability of testosterone and cortisol profiles was explained mainly by the PC2 (24.86%). In this case, the abovementioned variables were found on opposite sides of the PC2 axes. The variances of epitestosterone and corticosterone concentrations were accounted for mainly by the PC3 (17.74%). Both variables were positioned in the bottom part of the PCA plot. Thus, the first three PCs together explained 75.55% of the total variance of the analyzed data set. Moreover, it can also be noticed that steroid hormones were found in different positions on the PCA graph depending on the function in the human body. Thus, both glucocorticoids and androgens (testosterone and epitestosterone) were found on the right side of the PC1 axes, but their localizations on the PC2 axes were different, whereas the main naturally occurring human progestogen—progesterone was found as an outlier on the left of the PC1 axes.

As shown in Figure 4B, all the subjects studied were located within four clusters. Most of the healthy controls (15/20—75%) were positioned in clusters I and II while 18 out of 19 patients with NETs (94.7%) were found within clusters III and IV, respectively. Moreover, higher differences of hormone steroid profiles were observed for HC than those determined in patients with NETs. Notably, the patients with NETs treated with somatostatin analogue (PM: 2, 3, 6, 9, 10—cluster III and PF: 1–4, 8—cluster IV) were found together with those without somatostatin therapy (PM: 4, 5, 8—cluster III and PF: 5–7, 9—cluster IV), respectively. 

Only PM1 treated with somatostatin analogue was positioned in cluster II, but the unusually high concentration of cortisone (183.1 ng mL^−1^) for this patient was probably associated with methylprednisolone therapy when the cortisol level was low (16 ng mL^−1^). This type of therapy was not used for other subjects with NETs.

Moreover, the clustering of HC and P was mainly related to glucocorticoid profiles, the separation of men and women within both analyzed groups was mainly associated with progesterone and testosterone concentrations, and then epitestosterone profiles.

Thus, eight healthy men (HM: 1, 2, 4, 5, 7–10) identified against higher concentrations of testosterone, cortisone and cortisol on one hand, and lower progesterone and corticosterone levels on the other hand, were found in cluster I. Six healthy women (HF: 4–9) as well as HM6 and PM1 were located within cluster II. For them, the observed values of cortisol (with the exception of PM1), cortisone and corticosterone were high in contrary to low testosterone and varied progesterone levels. Eight men with NETs (PM: 2–6, 8–10) and HM3 characterized by lower cortisone and cortisol concentrations, but intermediate and higher epitestosterone profiles were placed within cluster III. Nine women with NETs (PF: 1–9) as well as four healthy women (HF: 1–3, 10) and PM7 described by intermediate values of cortisol and cortisone on one hand, and higher progesterone profiles on the other hand, were found in cluster IV. Moreover, it should be highlighted that better separation of HM against PM was found in comparison to that observed between PF and HF. This was probably related with greater diversity of cortisol and cortisone profiles in women than men. In consequence, the prognosis in NET disorders based on the quantification of cortisol and cortisone in urine may be considered for men, whereas in the case of women it may be difficult and ambiguous.

In summary, determinations of urinary glucocorticoid levels such as cortisol and cortisone could be considered in prognosing patients with NETs, which was also confirmed by the parametric and non-parametric test results. On the other hand, lower differences in glucocorticoid profiles between HF and PF as well as minor variations of epitestosterone levels for HC and P can sometimes be too difficult or risky for the correct interpretation of the experimental data. Thus, the extended application of the methods offering the determination of the metabolic profiles of the tested hormone levels and the metabolic syndrome for the prediction of hormone disturbances in correlation to the NET disorders should be continued. It should also be noticed that the use of chemometric techniques like PCA offers the possibility of a more detailed and deeper interpretation of the obtained results by taking into account the overall correlations as well as specific relationships between the variables and the subjects within analyzed data sets.

## 3. Experimental

### 3.1. Reagents

Glucocorticoids, including cortisol (11β,17α,21-trihydroxypregn-4-ene-3,20-dione), cortisone (4-pregnene-17α,21-diol-3,11,20-trione), corticosterone (4-pregnene-11β,21-diol-3,20-dione), and other hormones like testosterone (17-β-hydroxyandrost-4-en-3-one), epitestosterone (17-α-hydroxyandrost-4-en-3-one), progesterone and methyltestosterone (17β-hydroxy-17α-androst-4-ene-3-one) used as the internal standard (I.S.) were purchased from Sigma-Aldrich (St. Louis, MO, USA). All substances were of a minimum purity of 99%. HPLC grade acetonitrile and methanol as well as analytical grade acetone were obtained from Merck (Darmstadt, Germany). All chemicals and reagents were applied without further purification. Water used in all experiments was obtained from Milli-Q equipment (Millipore, Molsheim, France), whereas Supel-Select hydrophilic-lipophilic balance (HLB) cartridges (200 mg, 3 mL) used in solid-phase extraction (SPE) were supplied by Supelco (Park Belefonte, PA, USA).

Stock solutions of all analyzed hormones and methyltestosterone (I.S.) were prepared by dissolving (10.0 mg) each of the analytes in 10 mL of methanol. Standard working solutions were prepared daily in glass volumetric flasks by appropriately diluting stock solutions with methanol to obtain the final concentrations of 100, 10, 1 µg mL^−1^ as well as 100 and 10 ng mL^−1^ of the analyzed steroids. All stock and working solutions were stored in the dark at 4 °C until use to avoid possible decomposition. Calibration standard solutions containing 1, 5, 10, 20, 50, 100, 200, and 300 ng mL^−1^ of the compounds of interest and with the I.S. at a concentration of 100 ng mL^−1^ were prepared in charcoal-stripped urine and treated as described under sample preparation.

### 3.2. Sample Preparation

In the study, sample procedure preparation as described in Ref. [32] was used, where only one modification related to the use of methyltestosterone at a concentration of 100 ng mL^−1^ as the I.S., instead of dexamethasone, was introduced.

### 3.3. LC Conditions

LC analysis was performed with the Agilent 1260 Infinity system (Agilent Technologies, Santa Clara, CA, USA) consisting of a binary pump, an autosampler with an injector with a 30 µL sample loop volume, a UV variable wavelength detector and a computer system for data acquisition (ChemStation software; v. B.04.03, Agilent Technologies). Chromatographic separation of the analytes was carried out under gradient elution of solvent A (water) and solvent B (a mixture of acetonitrile and methanol, 50:50, *v/v*) using a Discovery^®^ HS C 18 column (250 × 4 mm, 5 μm) purchased from Supelco (Bellefonte, PA, USA). An optimized gradient program was realized: (*i*) 0–20 min, gradient elution formed from 5% to 100% B; (*ii*) 20.0–20.1 min, from 100% to 5% B; (*iii*) 20.1–30 min, 5% B to achieve column equilibration. The flow rate was maintained at 1 mL min^−1^, and the compounds were recorded with a UV detector set at a wavelength of 240 nm. The LC system was operated at a temperature of 40 °C. Under these conditions, the retention times for cortisone, cortisol, corticosterone, testosterone, epitestosterone, progesterone and the internal standard (methyltestosterone) were 14.73, 15.04, 16.71, 18.16, 18.90, 19.35 and 20.75 min, respectively. The total time of a single analysis, including equilibration of the column, was 30 min.

### 3.4. Validation of the Analytical Method

The validation of the method for the quantitation of six steroid hormones in human urine was performed according to the Food Drug Administration (FDA) and ICH guidelines.

### 3.5. Application of the Method in Real Human Urine Samples

The LC method was used to measure free endogenous levels of cortisol, cortisone, corticosterone, testosterone, epitestosterone and progesterone in real urine samples from 19 patients with diagnosed NETs (ten males and nine females) and from 20 healthy volunteers (ten males and ten females). Both the groups of patients with NETs and the healthy controls were matched according to age, gender and BMI. The study protocol was formally approved by the Ethical Committee from the Medical University of Gdańsk, and a written informed consent was obtained from each participant before entering the study. Thus, patients with NETs were aged between 35 and 73 years (mean ± standard deviation; 54.6 ± 11.8 years), weighing from 52 to 90 kg (68.7 ± 11.6 kg) and from 160 to 188 cm (171 ± 8 cm) in height, while healthy volunteers were aged between 33 and 75 years (47.3 ± 12.5 years), weighing from 55 to 90 kg (72.0 ± 11.2 kg) and from 154 to 190 cm (171 ± 10 cm) in height, respectively. Patients representing various stages of NET diseases and different therapies were hospitalized in the Department of Endocrinology at the Medical University of Gdansk (Gdansk, Poland). Prior to medical history data collection, a personal interview was conducted using a structural questionnaire. Participants were asked whether they had ever been diagnosed, by a physician, with kidney dysfunction and Cushing’s syndrome. Next, the medical history data included demographic characteristics, family history of NET disorders and other diseases as well as dietary habits, and all treated medications were collected. Among the analyzed patients with NETs, six persons were diagnosed with a pulmonary NET, six others with a carcinoid tumor of the small intestine with metastases to the lymph (5), the liver (4), and the bones (2), respectively. Moreover, a breast NET with metastases to the bones (1), a gastric NET with metastases to the liver, the lymph and the bones (1), a pancreatic NET with metastases to the liver and the lymph (2), a carcinoid of the appendix (1), as well as a NET of the rectum (1), and an unknown primary origin with metastases to the liver (1) were diagnosed for the patients studied.

The health status of each control subject was determined from his/her medical history, physical examination, and routine laboratory tests (blood chemistry, hematology and urine analysis). 24-h urine specimens from control subjects and the patients with NETs were collected and were stored in the dark at 4 °C during the sample collection. Next, the sample volume for each subject was measured, and 20 mL from each sample was placed in polyethylene urine containers at −80 °C until the LC analysis. Moreover, for each participant the measurement of creatinine was performed using a diagnostic kit for creatinine determinations from PZ Cormay (Lublin, Poland) according to the supplied methodology.

### 3.6. Data Treatment

A statistical analysis of the profile steroid levels, including cortisone, cortisol, corticosterone, testosterone, epitestosterone and progesterone, obtained from the patients with NETs and the control subjects participating in the study was performed using Statistica 9.1 software (StatSoft, Tulsa, OK, USA). This assay was conducted to evaluate the opportunity to consider steroid hormone levels as biomarkers of NETs.

#### 3.6.1. Statistical Analysis Based on Parametric and Non-Parametric Tests

The statistical analysis of differences between six urinary steroid levels obtained from the whole group of patients with NETs and the healthy volunteers (both males and females), as well as alternations of hormone concentrations observed for women and men determined within all the tested groups was performed using parametric and non-parametric tests.

At the beginning, all experimental data sets were evaluated by the Shapiro-Wilk test and Brown-Forsythe test for checking whether the variables met the criteria for normal distribution and homogeneity of variance, respectively. Both parameters were calculated for all the analyzed groups, with the significance at *p* < 0.05 (data not shown). If the above-described criteria were met for both compared groups, parametric analysis using the Student’s *t*-test was applied. If the normal distribution of the variable was confirmed but the homogeneity of variance was not proven, the separate-variances t-test was used. In the event of the failure of at least one from the compared groups to meet the criteria of normal distribution, the differences between the analyzed groups were evaluated by a non-parametric test, namely the Mann-Whitney U test. All tests were used at the significance level of 0.05. The parametric and non-parametric test results for all steroid profiles calculated for all groups are presented in Table 4. 

#### 3.6.2. PCA Analysis

Chemometric analysis, namely principal component analysis (PCA) based on the quantification of six urinary steroid levels was performed in order to visualize the differences between the tested objects (healthy controls and patients with NETs) described by many variables. The PCA loadings and score plots are shown in Figure 4A,B, respectively.

## 4. Conclusions

A reliable, accurate and specific LC method for the simultaneous determination of six steroid hormones in urine samples with UV detection has been developed. The usefulness of the proposed method as an alternative analytical tool in pharmacokinetic and biomedical studies was confirmed during the monitoring of free endogenous steroids in 20 healthy volunteers and 19 patients with neuroendocrine tumors. Moreover, a detailed statistical comparison of steroid hormone profiles from both the analyzed groups, including males and females separately, was performed using parametric and non-parametric tests, and principal component analysis. The obtained results lead to the conclusion that steroid profiles may create new possibilities for biomarker research and in the aspect for the prognosis in NETs. It was confirmed that the steroid profiles, especially cortisone and cortisol levels, were suitable factors in clinical practice for men, and could be recommended for clinical and biomedical investigations aimed at the discovery of hormonal disturbances and the related NETs. In the case of women, further study should be continued because the results were not consistent. Finally, an application of the presented method coupled with the use of many statistical tools for the analysis of the experimental data based on the quantification of steroid hormone profiles in human urine could be useful for the improvement of the follow-up of NET treatment.
